# Patchouli Alcohol Inhibits D-Gal Induced Oxidative Stress and Ameliorates the Quality of Aging Cartilage via Activating the Nrf2/HO-1 Pathway in Mice

**DOI:** 10.1155/2022/6821170

**Published:** 2022-06-08

**Authors:** Ming Chen, Haiyan Wen, Siqi Zhou, Xinxin Yan, Haohuan Li

**Affiliations:** ^1^Department of Orthopedics, Renmin Hospital of Wuhan University, Wuhan 430060, China; ^2^Department of Pharmacy, Renmin Hospital of Wuhan University, Wuhan 430060, China

## Abstract

Chondrocytes play an essential role in maintaining the structure and function of articular cartilage. Oxidative stress occurred in chondrocytes accelerates cell senescence and death, which contributes to the development of osteoarthritis (OA). Patchouli alcohol (PA), a kind of sesquiterpene in *Pogostemon cablin*, processes multiple bioactivities in treatment of many diseases. However, its effects of antisenescence and antioxidation on chondrocytes in a D-gal-induced aging mice model are still obscure. In this study, we found that PA treatment could ameliorate the degradation of cartilage extracellular matrix (ECM) in a D-gal-induced aging mice model. Further analyses through the immunofluorescent staining and western blot revealed that PA inhibited D-gal-induced chondrocyte senescence via the activation of antioxidative system. Besides, the damage caused by D-gal could not be recovered with PA treatment in Nrf2-silencing chondrocytes. In addition, molecular docking analysis between PA and Keap1 further suggested that the mechanism of PA's antisenescence and antioxidation was attributed to the activation of Nrf2/HO-1 pathway. Therefore, our results demonstrated that PA was a promising candidate for preventing the quality loss of aging cartilage through inhibiting oxidative stress-mediated senescence in chondrocytes.

## 1. Introduction

As a complex and unmanageable natural process in the human organism, senescence can be broadly defined as the time-dependent cumulation of cellular damage or stress influenced by the interplay of multiple genetic and environmental factors, which can result in the progressive decline of tissues and organ function [1, 2]. Senescence is regarded as one of important drivers in many diseases and has been linked to articular disease such as osteoporosis, macular degeneration, and osteoarthritis (OA) [3]. Chondrocytes, the main cell type in articular cartilage, produce extracellular matrix (ECM) and play an essential role in maintaining the structure and function of articular cartilage. It was reported that senescent chondrocytes were found in OA lesion, contributing to the development or progression of disease [4]. Normally, chondrocytes are hyporeplicative during homeostasis with limitation proliferation ability [5]. Senescent chondrocytes exhibit telomere shortening with age and the nonreplicative senescence of chondrocytes mainly induced by external or endogenous stress, such as reactive oxygen species (ROS) [6]. Previous studies have confirmed that extravagant levels of ROS could, in turn, aggravate senescence and set off pathological modifications of cellular proteins and lipids, thereby restraining chondrocyte activity and destroying ECM homeostasis, finally giving rise to damage of articular cartilage [7, 8]. Thus, inhibition of chondrocyte oxidative stress is proposed as an effective remedy strategy for senile cartilage quality loss.

The Kelch-like ECH-associated protein 1 (*Keap1*)–nuclear factor-erythroid 2-related factor 2 (*Nrf2*)-antioxidant response element (ARE) system is one of the uppermost reaction mechanisms to antioxidative stress damage in the aging process [9]. This defensive system is regulated by Nrf2 and its repressor Keap1, and its disruption might promote stress-induced premature senescence [10]. In response to oxidative stress, Nrf2 is decoupled from cytosolic repressor protein Keap1 and transfers to the nucleus, binding with the ARE and subsequently activating downstream related gene transcriptions, such as catalase (CAT), heme oxygenase-1 (HO-1), superoxide dismutase (SOD), glutathione (GSH), and other cytoprotective enzymes, thereby preventing the cells from oxidative stress damage [11, 12]. Liu et al. isolated a new type of bioactive peptide F2d from rice residue, which acted on umbilical vein endothelial cells and exerted antioxidant stress function through promoting Nrf2 signal pathway [13]. A recent study found that a small molecule SR9009 blocks cellular senescence via the Nrf2 pathway *in vitro* and *in vivo* [14]. Therefore, increased Nrf2 activation is an efficient therapeutic tactic that arouses integral antioxidant capacity and attenuates aging.

D-galactose- (D-gal-) induced aging animal model has been widely applied to study the senescence mechanism and antiaging efficacy of drugs [15]. It is considered as an agent for inducing animal senescence for its ability to accumulate ROS, which further brings about a physiological state similar to aging, consisting of decreased antioxidant activity, increased free radical level, and enhanced oxidative stress level in all organs of the organism [16, 17]. *Pogostemon cablin* is a Chinese herbal medicine with the functions of dispelling dampness and removing heat and external syndrome [18]. It has also been currently devoted to treating exogenous fever, vomiting, liver injury, and intestinal barrier impairment [19]. Patchouli alcohol (PA), the main bioactive component of *Pogostemon cablin*, has been found to have a variety of biological activities such as anti-inflammatory, antisteatosis, antioxidant, and immunomodulatory [20, 21]. Besides, recent studies have validated that PA markedly suppressed ROS levels and increased antioxidant enzyme activities via the Cyp2e1/ROS/Nrf2/HO-1 pathway to ameliorate acute liver injury [22]. Nevertheless, the antioxidant stress and antisenile cartilage quality reduction effects of PA in D-gal-induced aging models remain to be further confirmed.

Hence, in this work, we used chondrocytes induced by D-gal and D-gal-mediated mouse aging model to study the protective effect and potential mechanism of PA on low quality of senile cartilage. In the mice aging model, we found that PA could significantly alleviate aging symptoms and reverse the decline in aging cartilage quality induced by D-gal. Further studies showed that PA treatment relieved cytotoxicity, oxidative stress, senescence, and ECM catabolism in chondrocytes induced by D-gal *in vitro*. In addition, RNA interference assay (RNAi) and molecular docking of PA with Keap1 protein were implemented to elucidate the mechanism of PA aging-delaying. Here, our study suggested that PA could effectively alleviate D-gal-induced aging and suppress oxidative stress via Nrf2/HO-1 pathway in vitro and in vivo.

## 2. Materials and Methods

### 2.1. Materials and Reagents

Patchouli alcohol (PA) (purity ≥98%) was purchased from Mansite Biotechnology Co., Ltd. (Chengdu, China). PA was dissolved in dimethyl sulfoxide (DMSO) for subsequent cell treatment (final DMSO concentration did not exceed 0.1% in all experiments). D-gal (purity ≥98%) was purchased from Kerui Biotechnology Co., Ltd. (Wuhan, China). The assay kits for MDA, SOD, GSH, and CAT were obtained from Jiancheng Bioengineering Institute (Nanjing, China). The DAB chromogenic kit and DAPI staining solution were obtained from Servicebio (Wuhan, China). The Dulbecco's modified eagle medium-F12 (DMEM) and fetal bovine serum (FBS) were obtained from HyClone Co. (Logan, USA), and 1% penicillin/streptomycin was ordered from Servicebio (Wuhan, China). The antibody for collagen II (COL2A1, GB11021) and HO-1(GB11104) was obtained from Servicebio (Wuhan, China). The antibodies against glyceraldehyde phosphate dehydrogenase gene (GAPDH, A19056), lamin B (A1910), aggrecan (ACAN, A8536), a disintegrin and metalloproteinase with thrombospondin 5 (ADAMTS5, A2836), and matrix metalloproteinase 13 (MMP13, A11148) were obtained from Abclonal (Wuhan, China). The antibodies of TP53 (AF0879), Nrf2 (AF7006), and Cyclin-Dependent Kinase Inhibitor 1A (CDKN1A/p21^Cip1/Waf1^, AF6290) were purchased from Affinity Biosciences Ltd. (Cincinnati, USA). All primers were obtained from Tianyi Biotech Co., Ltd. (Wuhan, China).

### 2.2. Animals and Experimental Design

The specific pathogen free (SPF) C57BL/6 mice (No. 2020-0018, license number: SCXK (Hubei), certification number: 42000600040335) were purchased from the Experimental Center of the Hubei Medical Scientific Academy (Wuhan, China). The Committee on the Ethics of Animal Experiments of the Wuhan University School of Medicine approved the protocol (Permit 14016). All experimental animal procedures were conducted following the Guidelines for the Care and Use of Laboratory Animals of the Chinese Animal Welfare Committee. Healthy animals weighing 25–31 g and aged 10–12 weeks were housed in an air-conditioned room under standard conditions (room temperature, 20–23°C; humidity, 50–60%; and light, 12 h light/dark cycle), where standard laboratory chow and water were freely consumed. To use for reference, the D-gal aging mice model was constructed by the previous research [23]. In simple terms, after 1 week of adaptive feeding, the animals were randomly divided into 3 groups (n = 8 each group), namely, the NOR, D-gal, and D-gal and PA groups. The mice in the D-gal and D-gal and PA groups were subcutaneously injected with D-gal (150 mg/kg) for 8 consecutive weeks to induce senescence, while the NOR group received subcutaneous injection of an equivalent dose of normal saline. Based on the initial treatment, beginning at the fourth week, the mice in the D-gal and PA group were intraperitoneally administered with PA (20 mg/kg) [20]. The unilateral knee specimens were collected and fixed with 4% paraformaldehyde for 48 h for further histological analysis.

### 2.3. Histopathological Evaluation

The mice articular cartilage tissues were fixed in 4% paraformaldehyde for 48 h, embedded in paraffin, and cut into slices, which were processed and stained with alcian blue and safranin O-fast green (SO) to visualize the smoothness of cartilage surface and proteoglycan content (five sections in each group). The modified Mankin's scoring system was used to quantify the degeneration of cartilage [24].

For immunohistochemistry and immunofluorescent staining, five cartilage slices in each group were subjected to staining of COL2A1, TP53, and Nrf2. Firstly, the cartilage slices were antigenically repaired in citric acid repair buffer (pH 6.0) for 6-8 hours at 60°C. Subsequently, endogenous peroxidase activity was sealed by treating with 3% H_2_O_2_ for 25 min, and then, sections were blocked in 3% BSA at 37°C for 50 min. After incubation with the primary antibodies, the sections were incubated with goat anti-rabbit IgG secondary antibody (1 : 100, Abclonal, China) or Alexa Fluor-conjugated secondary antibody (1 : 100; Abclonal, China). Immunofluorescence sections could be sealed for observation after DAPI staining for 8 min, while immunohistochemistry required DAB chromogenic reaction and nuclear treatment with hematoxylin. In the end, the representative images were captured under a Nikon NIS Elements BR light microscope (Nikon, Japan).

### 2.4. Isolation and Primary Culture of Chondrocytes

The articular cartilage of 2-week-old C57BL/6 mice was separated and cut into 1 mm^3^ piece under sterile conditions and then digested with 2 mg/ml of 0.1% collagenase II at 37°C for 6-8 h. The digested cartilage tissue was centrifuged, suspended in cartilage growth medium (containing 10% fetal bovine serum, DMEM/F12 medium, and 1% penicillin/streptomycin), and seeded into tissue culture flasks. The chondrocytes were cultured at 37°C under 5% CO_2_ for 24 h, then the medium was changed, and the second- or third-generation cells were taken for subsequent experiments.

### 2.5. Cell Viability

Cell Counting Kit-8 (CCK-8) was constantly used to assess the viability of cells. In brief, chondrocytes inoculated to 96-well plates were treated with PA (0, 2.5, 5, 10, 20, and 40 *μ*M) or D-gal (0, 2.5, 5, 10, 15, and 30 mg/ml) for 24 h at 37°C [20, 25]. After that, 10 *μ*l of CCK-8 solution was added to the cell wells and incubated at 37°C for 40 min. Finally, the light absorbance reflecting the cell viability at 450 nm was detected by fluorescence microplate (Olympus, Japan).

### 2.6. Analysis of Oxidative Stress Markers and Enzyme Activities

The fresh blood of mice was quickly obtained before sacrifice and centrifuged at 5000 rpm and 4°C for 15 min, and then, the supernatant was absorbed and stored at -80°C. At the same time, all organs were dissected and weighed and then kept at −80° C. In addition, the organ index was calculated with the following formula: organ index = organ weight (mg)/body weight (g). Afterwards, the activities or levels of CAT, SOD, GSH, and malondialdehyde (MDA) were determined with commercial kits in the light of the manufacturer's instructions. For the detection of SOD and GSH in chondrocytes, we collected cells for centrifugation and ultrasonic fragmentation and then carried out subsequent operations according to the instructions.

### 2.7. Cytopathic Staining

The chondrocytes were seeded in 12-well plates and preincubated with PA at concentrations of 0, 2.5, 5, and 10 *μ*M for 24 h and then exposed with D-gal (5 mg/ml) for 24 h. The alcian blue and safranin O staining procedures were performed: after rinsing thrice with PBS, the chondrocytes were fixed with 4% paraformaldehyde for 15 min and stained with 1.0% alcian blue solution or 0.5% safranin O solution for 30 min at room temperature. At last, the cytopathic staining solutions utilized to the steady chondrocytes were removed and washed by PBS three times for 5 min each. The activity of *β*-galactosidase (bright blue cells are considered as positive) was determined by utilizing SA-*β*-Gal staining kit (Beyotime, China) on the basis of operating guide. The photomicrographs of the stained chondrocytes were obtained by a Nikon NIS Elements BR light microscope (Nikon, Japan).

### 2.8. ROS Measurements and Immunofluorescence

ROS produced by D-gal (5 mg/ml) or PA (10 *μ*M) disposal in chondrocytes was evaluated by fluorescence intensity of the 2′,7′-dichlorodi-hydrofluorescein diacetate (DCFH-DA) probe. Following the treatment of D-gal or PA, chondrocytes were rinsed and incubated with 5 mg/ml DCFH-DA probe (Beyotime, China) for 40 min in 37°C darkness. After washing, fluorescent signals were immediately measured using a fluorescence microscope (Nikon, Japan). Besides, the chondrocytes were inoculated on the cover glass and treated with PA (10 *μ*M) or D-gal (5 mg/ml) for 24 h. After PBS washing, the slices were fixed with 4% paraformaldehyde for 15 min, washed with PBS, and permeabilized with 0.5% Triton X-100 for 15 min at room temperature. After 50 min of 5% bovine serum albumin incubation, the slices were rinsed with PBS and treated with primary antibodies overnight at 4°C. In the next day, the slices were incubated with Alexa Fluor-conjugated secondary antibody (1 : 100; Abclonal, China) in the dark for 1 h and then stained with DAPI (Servicebio, China) for 8 min. At last, the representative images were captured under a fluorescence microscope (Nikon, Japan).

### 2.9. Gene Expression

Total RNA was extracted from cartilage tissue and BMSCs with TRIzol reagent (Life Technologies, USA). The concentration and purity of extracted RNA were detected by a spectrophotometer (Thermo, USA), and finally the RNA concentration was modulated to 1 *μ*g/*μ*l. Then, the total RNA was reverse-transcribed into cDNA with the help of a Transcriptor First Strand cDNA Synthesis Kit (Vazyme, China) according to the manufacturer's enchiridion. Subsequently, the cDNA was mixed with SYBR Green Supermix (Servicebio, China) and the primers (Table 1) utilizing the ABI Step One RT-PCR thermal cycler (ABI StepOne, USA) in a 10 *μ*l reaction mixture for real-time quantitative PCR (RT-qPCR). In the end, the 2^-*ΔΔ*Ct^ method was employed to determine relative mRNA expression level, and the GAPDH gene was used as a reference value to normalize.

### 2.10. Western Blot Analysis

The chondrocytes were preincubated with PA at concentrations of 0, 2.5, 5, or 10 *μ*M for 24 h and then exposed with D-gal (5 mg/ml) for 24 h. Subsequently, the chondrocytes were lysed by radioimmunoprecipitation assay (RIPA) buffer (Beyotime, China) containing a mixture of protease and phosphatase inhibitors. Protein samples were subjected to SDS-PAGE and transferred to PVDF membranes (Bio-Rad, USA). The membranes were blocked by 5% skim milk for 1 h and incubated overnight at 4°C with the appropriate primary antibodies, respectively. On the following day, after washing with TBST for 30 min, the membranes were incubated with HRP-conjugated secondary antibodies (1 : 5000, Abclonal, China) for 1 h at room temperature. The enhanced chemiluminescence method was conducted to observe the bands, and ImageJ software was applied for quantitative analysis.

### 2.11. Nrf2 siRNA

To knockdown Nrf2 expression, the siRNA oligonucleotides against mice Nrf2 were purchased from GenePharma Co., Ltd. (Shanghai, China). Nrf2 siRNA or the negative control siRNA transfection was performed according to the manufacturer's instructions of Lipofectamine 3000 reagent (Invitrogen, USA). The chondrocytes were seeded in 96-well plates or 6-well plates with siRNA transfections (50 nM) for 48 hours, and the chondrocytes were predisposed with PA (10 *μ*M) for 24 h, followed by incubating with D-gal (5 mg/ml) for 24 h, and then harvested for follow-up experiments.

### 2.12. Molecular Docking

The molecular structures of Keap1 inhibitor KI-696^26^ (a chemically synthetic ligand that has been reported as Keap1 inhibitor), theaflavin [26] (a natural compound derived from black tea that was reported as an activator of the Nrf2 pathway), and PA were downloaded from PubChem (https://pubchem.ncbi.nlm.nih.gov/), and the energy of these ligands was minimized by Chem3D 19.0. The Keap1 protein (ID: 1X2J) was downloaded from the PDB database (http://www.rcsb.org). Then, KI-696, theaflavin, and PA were docked into 1X2J by Autodock 4.2.6, respectively. The default values are used for all parameters. Finally, the lowest energy conformations were chosen and visualized by virtue of PyMOL software (version 2.3.3) and Ligplot^+^ (version 2.2.4).

### 2.13. Statistical Analysis

GraphPad Prism 8.0 software was applied for statistical processing. The results are presented as relative ratios compared with the control values (mean ± SEM). *T*-test or one-way analysis of variance (ANOVA) was applied for comparison between two or more groups, and Tukey posttest was employed for comparison within multiple groups. All experiments were performed at least three times. *P* < 0.05 was regarded statistically significant.

## 3. Results

### 3.1. Effects of PA on Mental State, Body Weight, and Organ Indices

Through observation, we found that mice in the D-gal group showed dull hair, lethargy, and slow reaction, while mice in the NOR group were active and sensitive to sound and light stimulation and have white and shiny hair. Meanwhile, the mice in the D-gal group showed a loss in the body weight and organ indices compared with the NOR group (Table 2). However, the poor mental state, body weight loss, and organ index decline of mice in the D-gal group were alleviated to a certain extent after 4 weeks of PA treatment.

### 3.2. PA Reverses the Decline in Gerontal Cartilage Quality in D-Gal-Caused Mice

**Figure 1:**
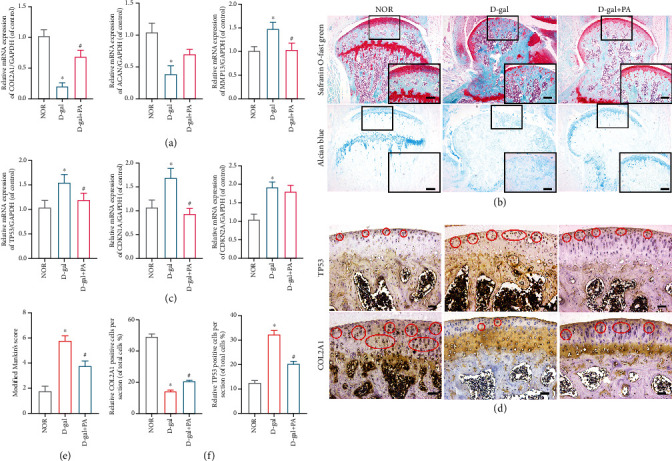
Influences of patchouli alcohol (PA) on cartilage quality in D-gal-induced aging mice. (a) Effect of PA on ECM degradation marker gene expression of collagen 2a1 (COL2A1), aggrecan (ACAN), and matrix metalloproteinase 13 (MMP13) in D-gal-caused senescent chondrocytes was analyzed by RT-qPCR. (b) The quantitative real-time PCR (qRT-PCR) results of the mRNA expression of Tp53, Cyclin-Dependent Kinase Inhibitor 1A (Cdkn1a/p21^Cip1/Waf1^), and Cyclin-Dependent Kinase Inhibitor 2A (Cdkn2a/p16^INK4a^). (c) Representative alcian blue and safranin O-fast green (SO) staining images in NOR group, D-gal group, and D-gal and PA (20 mg/kg) group. Scale bar, 250 *μ*m; scale bar (enlarged), 100 *μ*m. (d) Immunochemical staining of articular cartilage with COL2A1 and MMP13 in the NOR group, D-gal group, and D-gal and PA (20 mg/kg) group (staining positive areas were circled in red). Scale bar, 100 *μ*m. (e) The diagram was applied to display modified Mankin's score of articular cartilage. (f) Quantitative analysis of COL2A1 and MMP13 immunostaining (positive cells %). Scale bar: 50 *μ*m. Mean ± SEM. ^∗^*P* < 0.05 vs. NOR group; ^#^*P* < 0.05 vs. D-gal group.

In the first place, we evaluated the protective effect of PA (20 mg/ml) in D-gal (150 mg/kg) mice *in vivo*. The PA-treated mice had significantly shorter ECM degradation marker gene expression of COL2A1, MMP13, and ACAN in contrast to the mice in the D-gal group, while PA partially reversed ECM dysfunction in D-gal-induced aging mice (Figure 1(a)). Further, according to the results of SO and alcian blue staining, some meaningful cartilage lesions were noticed in the D-gal group in comparison to the NOR group, such as chondrocyte decrease, an uneven cartilage surface, Mankin's score increase, cartilage tissue reduction, and proteoglycan loss (Figures 1(b) and 1(e)). In contrast, more chondrocytes, more proteoglycans, smoother articular surfaces, and lower Mankin scores were observed in the D-gal and PA group compared to the D-gal group. In addition, the increased mRNA expression of aging marker genes (Tp53, Cdkn1a/p21^Cip1/Waf1^, and Cdkn2a/p16^INK4a^) suggested that D-gal treatment could lead to enhanced aging of articular cartilage, and PA treatment could partially alleviate the senescence induction effect of D-gal (Figure 1(c)). As shown in Figures 1(d) and 1(f), immunochemical staining demonstrated that PA could attenuate the decrease in the expression of COL2A1 in the cartilage of D-gal mice as well as suppressed the increase in the expression of TP53. Thus, these findings indicated that PA plays a protective role against the cartilage lesions and increased expression of aging markers *in vivo.*

### 3.3. PA Ameliorates Oxidative Stress and Activates Nrf2/HO-1 Signaling Pathway in D-Gal-Induced Aging Mice

**Figure 2:**
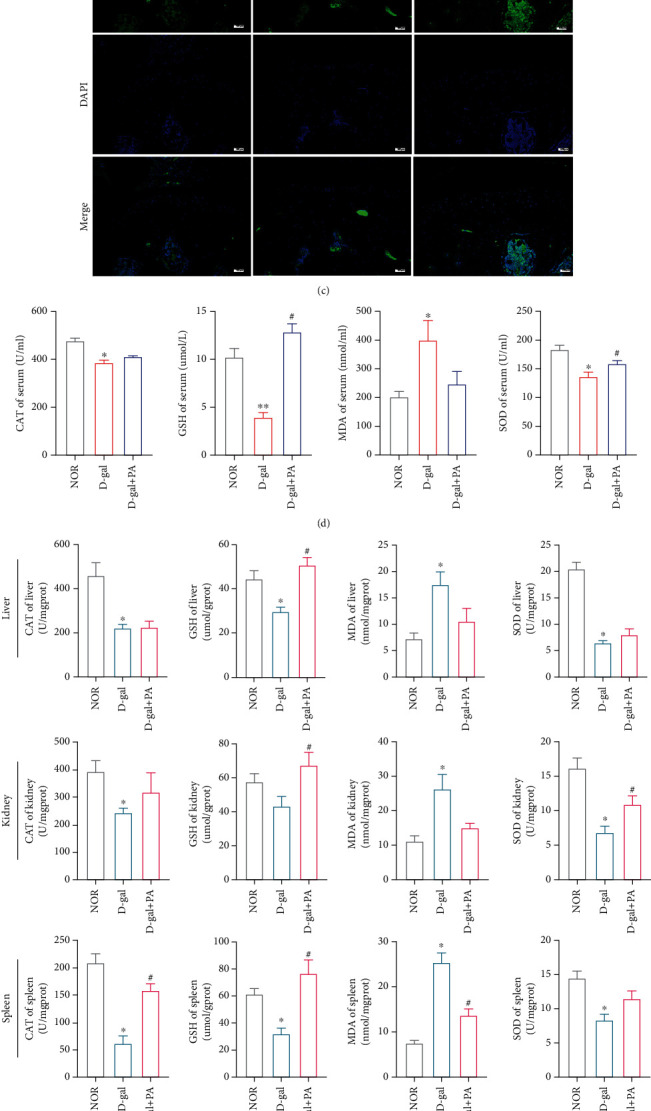
Influences of patchouli alcohol (PA) on oxidative stress and Nrf2/HO-1 signaling pathway in D-gal-induced aging mice. (a) The mRNA expression of Kelch-like ECH-associated protein 1 (Keap1) and nuclear factor-erythroid 2-related factor 2 (Nrf2) in knee joint cartilage from aging mice given PA. (b) The mRNA relative abundance of catalase (Cat) and superoxide dismutase 1 (Sod1) in D-gal-mediated senescence mice. (c) The expression of Nrf2 was evaluated using immunofluorescent staining. Scale bar, 100 *μ*m. (d) The activities or contents of serum oxidation activity indicators such as CAT, glutathione (GSH), malondialdehyde (MDA), and SOD were determined using commercial kits. (e) The activities or contents of CAT, GSH, MDA, and SOD in different organs (liver, kidney, spleen, and thymus) were determined by commercial kits. Mean ± SEM. ^∗^*P* < 0.05 and ^∗∗^*P* < 0.01 vs. NOR group; ^#^*P* < 0.05 vs. D-gal group.

Considering that PA plays a role in D-gal-mediated senile cartilage quality loss, it was speculated that the effects of PA could be, in part, due to the regulation of Nrf2/HO-1 pathway to inhibit chondrocyte oxidative stress. Thus, we first determined the gene expression levels of Nrf2 and Hmox1 in D-gal-induced senescence mice; the gene expression of Nrf2 and Hmox1 in articular cartilage was decreased in D-gal-treated mice in comparison with those in NOR group mice, while this inhibitory action was recovered by the PA (20 mg/kg) treatment (Figures 2(a) and 2(c)). And then, we evaluated the effect of PA on D-gal-induced oxidative stress; the decreased gene expression of antioxidant indices (Cat and Sod1) caused by D-gal could also be reversed by PA (20 mg/kg) administration (Figure 2(b)). At the same time, the activity or content of oxidation activity indicators including CAT, GSH, SOD, and MDA (MDA is often considered as the harmful substance produced by lipid peroxidation) in serum and organs (liver, kidney, spleen, and thymus) were also consistent with the above results (Figures 2(d) and 2(e)). Hence, we initially determined the positive effects of PA on oxidative stress and Nrf2/HO-1 signaling pathway in the D-gal aging model.

### 3.4. PA Ameliorates D-Gal-Induced Chondrocyte Senescence and ECM Homeostasis Imbalance *In Vitro*

**Figure 3:**
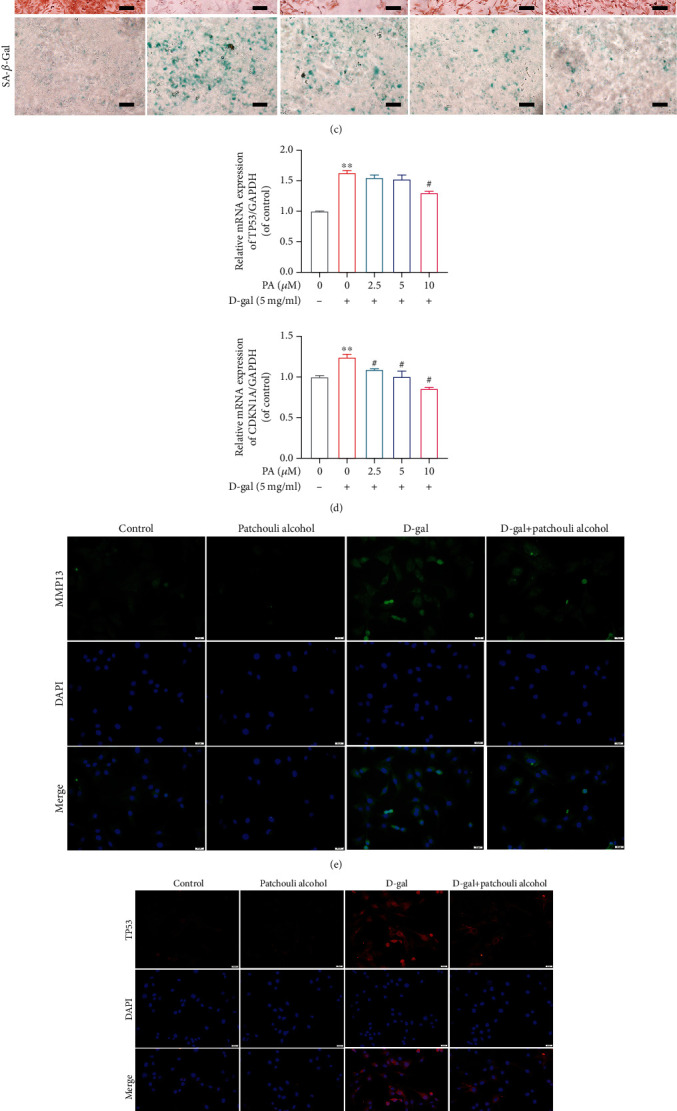
Influences of patchouli alcohol (PA) on senescence process and extracellular matrix (ECM) homeostasis of chondrocytes induced by D-gal. (a) Effect of PA on cell viability and cytotoxicity was determined by CCK-8 assay. (b) Effect of PA on ECM degradation marker gene expression of collagen 2a1 (Col2a1) and matrix metalloproteinase 13 (Mmp13) in D-gal-caused senescent chondrocytes was analyzed by RT-qPCR. (c) Representative images of alcian blue, safranin O, and SA-*β*-gal staining in chondrocytes treated with PA (0, 2.5, 5, and 10 *μ*M) and D-gal (5 mg/ml) for 24 h. The scale bar of alcian blue and safranin O staining, 250 *μ*m. The scale bar of SA-*β*-gal staining, 10 0 *μ*m. (d) Influence of PA on gene expression of Tp53 and Cyclin-Dependent Kinase Inhibitor 1A (Cdkn1a/p21^Cip1/Waf1^) in D-gal-caused senescent chondrocytes. (e and f) The immunofluorescence detection of MMP13 and TP53 in D-gal-caused senescent chondrocytes was treated with 10 *μ*M PA for 24 h. Scale bar, 50 *μ*m. (g) The expression and quantitative analysis of aggrecan (ACAN), COL2A1, a disintegrin and metalloproteinase with thrombospondin 5 (ADAMTS 5), and MMP13 proteins in chondrocytes disposed with PA (0, 2.5, 5, and 10 *μ*M) and D-gal (5 mg/ml) for 24 h were determined by western blotting. The protein expression level is the gray value ratio of the target protein to *β*-actin. (h) The expression and quantitative analysis of TP53 and CDKN1A/p21^Cip1/Waf1^ proteins in D-gal-mediated aging chondrocytes administrated with 10 *μ*M PA for 24 h. The protein expression level is the gray value ratio of the target protein to *β*-actin. Mean ± SEM. ^∗^*P* < 0.05 and ^∗∗^*P* < 0.01 vs. control group; ^#^*P* < 0.05 vs. D-gal group.

The D-gal-induced poor quality of age-related articular cartilage could often be owing to chondrocyte senescence and ECM homeostasis imbalance. Firstly, the CCK-8 assay was performed to evaluate the toxic effects of PA and D-gal on chondrocytes at different concentrations (Figure 3(a)). As shown in Figure 3(a), the 5 mg/ml D-gal intervention reduced chondrocyte viability, so 5 mg/ml D-gal was selected to establish a senescence model *in vitro*. In addition, pretreatment with PA (0, 2.5, 5, 10, and 20 *μ*M) reversed the decrease in the viability of chondrocytes induced by D-gal (Figure 3(a)). Secondly, we examined the effects of PA treatment at different concentrations (0, 2.5, 5, and 10 *μ*M) on D-gal (5 mg/ml) induced chondrocyte senescence and ECM homeostasis. After preprocessing mouse chondrocytes with PA for 24 h, the mRNA expression promotion of MMP13, TP53, and CDKN1A/p21^Cip1/Waf1^ and inhibition of COL2A1 induced by D-gal (5 mg/ml) were reversed (Figures 3(b) and 3(d)). Meanwhile, the results of safranin O and alcian blue staining reflecting ECM homeostasis showed that compared with the control group, the density and color of chondrocytes were diminished in D-gal group, and PA treatment could reverse this inhibition effect; the results of SA-*β*-gal staining reflecting chondrocyte senescence showed that the increased number of positive senescent cells caused by D-gal could also be reversed by PA administration (Figure 3(c)); the results of protein level reflecting degradation of ECM (COL2A1, MMP13, ACAN, and ADAMTS) were also consistent with the above results (Figures 3(e) and 3(g)). The 10 *μ*M concentration of PA selected for subsequent verification of senescence protein levels would follow shortly. The immunofluorescence or WB results indicated that the increased expression of senescence marker genes (Tp53 and Cdkn1a/p21^Cip1/Waf1^) in comparison with the control group could be reversed under PA (10 *μ*M) treatment (Figures 3(f) and 3(h)). These results demonstrated that PA inhibited the chondrocyte senescence and catabolism of the ECM induced in the chondrocytes by D-gal.

### 3.5. PA Attenuates D-Gal-Induced Oxidative Stress and Upregulates Expression of Nrf2/HO-1 Signaling Pathway of Chondrocytes *In Vitro*

**Figure 4:**
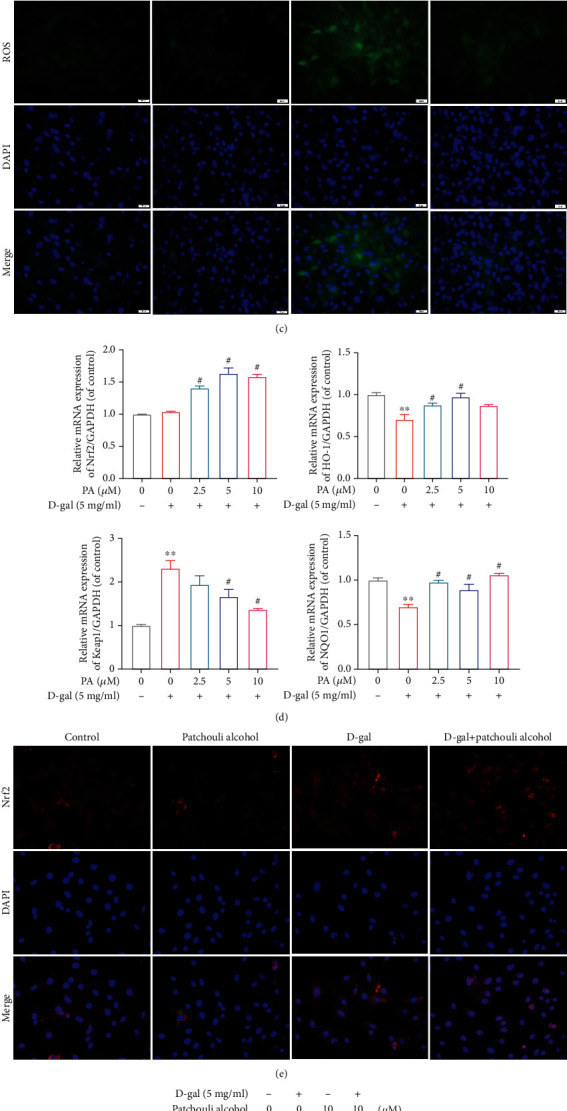
Influences of patchouli alcohol (PA) on oxidative stress and expression of Nrf2/HO-1 signaling pathway of chondrocytes mediated by D-gal. (a) Effect of PA on gene expression of catalase (Cat), glutathione synthetase (Gss), and superoxide dismutase 1 (Sod1) in D-gal-caused senescent chondrocytes was analyzed by RT-qPCR. (b) PA's effects upon antioxidant indices (CAT and SOD) generation by chondrocytes administrated with D-gal (5 mg/ml) and PA (0, 2.5, 5, and 10 *μ*M) for 24 h were determined by biochemical kit. (c) Representative fluorescent images of ROS levels in D-gal (5 mg/ml) induced senescent chondrocytes treated with 10 *μ*M PA for 24 h. Scale bar, 50 *μ*m. (d) The RT-qPCR was employed to detect the gene expression of Nrf2/HO-1 pathway, including nuclear factor-erythroid 2-related factor-2 (Nrf2), heme oxygenase-1 (Hmox1), NAD(P)H quinone dehydrogenase 1 (Nqo1), and Kelch-like ECH-associated protein 1 (Keap1), mediated by D-gal in aging chondrocytes. (e) Representative immunofluorescence pictures of Nrf2 were captured in D-gal (5 mg/ml) induced senescent chondrocytes. Nrf2 was stained with Nrf2-specific antibody (red staining), while the nucleus was stained with DAPI (blue staining). Scale bar, 50 *μ*m. (f) The expression and quantitative analysis of Nrf2 protein in the nucleus and HO-1 protein in the cytoplasm were assessed through western blotting. The Nrf2 and HO-1 protein expression levels are the gray value ratio of the target protein to lamin B and *β*-actin, respectively. Mean ± SEM. ^∗^*P* < 0.05 and ^∗∗^*P* < 0.01 vs. control group; ^#^*P* < 0.05 vs. D-gal group.

In order to better validate the role of PA in D-gal-induced oxidative stress injury and Nrf2/HO-1 pathway regulation, we conducted cell experiments to verify relevant indicators *in vitro*. In terms of gene expression levels, antioxidant stress indices (Cat, glutathione synthetase (Gss), and Sod1) and Nrf2/HO-1 pathway-related indicators (Nrf2, Hmox1, and NAD(P)H quinone dehydrogenase 1 (Nqo1)) were increased under PA (2.5, 5, and 10 *μ*M) treatment compared with D-gal (5 mg/ml) group (Figures 4(a) and 4(d)). The results of SOD and GSH enzyme detection in chondrocytes also supported the above phenomenon (Figure 4(b)). As illustrated in Figure 4(c), the chondrocytes exposed to D-gal (5 mg/ml) showed a significantly stronger ROS fluorescence signal than those of the control group sample, while PA (10 *μ*M) disposal reduced ROS levels. As mentioned before, Nrf2 is a transcription factor and uncoupled Nrf2 translocates into the nucleus to activate the transcription of target genes, including Hmox1. Based on the immunofluorescence results, we found that PA (10 *μ*M) disposal significantly promoted the nuclear translocalization of Nrf2 compared with the D-gal treatment group (Figure 4(e)). Further, our results from WB assay showed that the nuclear abundance of Nrf2 and cytoplasmic content of HO-1 decreased within 24 h after D-gal (5 mg/ml) administration in contrast with the control group, whereas PA (10 *μ*M) treatment partly recovered Nrf2 and HO-1 expressions (Figure 4(f)). These results indicated that PA attenuated D-gal-induced oxidative stress and upregulated expression of the Nrf2/HO-1 signaling pathway *in vitro*.

### 3.6. PA Acted against D-Gal-Induced Chondrocyte Senescence and ECM Degradation by Activating Nrf2/HO-1 Signaling Pathway *In Vitro*

**Figure 5:**
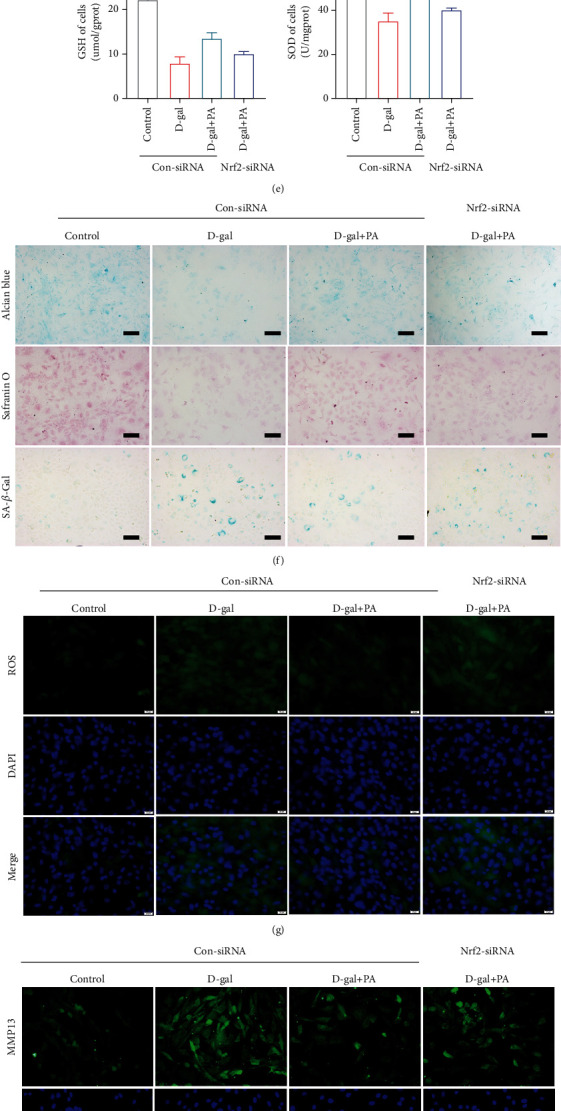
The suppressive effects of patchouli alcohol (PA) on D-gal-induced chondrocyte senescence via upregulating Nrf2/HO-1 signaling pathway. (a–c) Impact of PA on mRNA expression of collagen 2a1 (Col2a1), matrix metalloproteinase 13 (Mmp13), Tp53, Cyclin-Dependent Kinase Inhibitor 1A (Cdkn1a/p21^Cip1/Waf1^), catalase (Cat), and superoxide dismutase 1 (Sod1) in D-gal-caused senescent chondrocytes was detected by RT-qPCR. (d) Effect of PA on cell viability and cytotoxicity was determined by CCK-8 assay. (e) PA's influence upon antioxidant indices (CAT and SOD) generation by D-gal-induced senescent chondrocytes was determined by biochemical kit. (f) Representative images of alcian blue, safranin O, and SA-*β*-gal staining in aging chondrocytes. The scale bar of alcian blue and safranin O staining, 250 *μ*m. The scale bar of SA-*β*-gal staining, 100 *μ*m. (g) Representative fluorescent images of ROS levels in D-gal (5 mg/ml) induced senescent chondrocytes. Scale bar, 50 *μ*m. (h and i) The immunofluorescence detection of MMP13 and TP53 in D-gal-caused senescent chondrocytes. Scale bar, 50 *μ*m. (j) The expression and quantitative analysis of COL2A1, MMP13, nuclear factor-erythroid 2-related factor-2 (Nrf2), heme oxygenase-1 (HO-1), TP53, and p21 proteins were assessed through western blotting. The Nrf2 protein expression level is the gray value ratio of the target protein to lamin B, while other proteins were the grayscale ratio of target protein to *β*-actin. Mean ± SEM. ^∗^*P* < 0.05 and ^∗∗^*P* < 0.01 vs. corresponding group.

To further verify that Nrf2/HO-1 pathway was the main player in the beneficial effect of PA in the senescent chondrocytes and ECM degradation, the effect of Nrf2-siRNA coadministration on D-gal-induced chondrocytes was determined. The RT-qPCR results indicated that Nrf2-siRNA had a significant inhibition effect on mRNA expression of Col1a1, Cat, and Sod1 in response to D-gal (5 mg/ml) stimulation after PA (10 *μ*M) treatment compared to Con-siRNA but remarkably facilitated the expression of Mmp13, Tp53, and Cdkn1a/p21^Cip1/Waf1^ (Figures 5(a)–5(c)). The cell viability results suggested that the single treatment of Con-siRNA or Nrf2-siRNA had no obvious effect on cell viability; the cell viability of Con-siRNA-treated cells and Nrf2 knockdown cells was distinctly decreased after D-gal administration; the cell viability in Nrf2-siRNA-treated cells could not be recovered with PA treatment from the damage caused by D-gal (Figure 5(d)). Furthermore, the test of ROS levels and biochemical indicators of antioxidant stress (GSH and SOD) in chondrocytes indicated that compared with Con-siRNA-treated cells, the PA (10 *μ*M) disposal in Nrf2-siRNA-treated cells had not alleviated high oxidative stress viability mediated by D-gal (5 mg/ml) (Figures 5(e) and 5(g)). For degradation of ECM and chondrocyte senescence, the results of cytopathic staining and MMP13 and TP53 immunofluorescence detections showed that the exposure of Nrf2-siRNA-transfected cells to PA (10 *μ*M) and D-gal (5 mg/ml) resulted in a decrease of density and color of chondrocytes in alcian blue and safranin O staining in comparison with Con-siRNA group and an increase in the fluorescence signal of TP53 and MMP13 proteins and number of positive senescent cells (Figures 5(f), 5(h), and 5(i)). Moreover, the WB analysis assay in chondrocytes treated with Nrf2-siRNA further supported the above results (Figure 5(j)). Together, these results illustrated that the involvement of Nrf2/HO-1 in the protective effect of PA on antioxidant stress, chondrocyte senescence, and ECM degradation in mice chondrocytes.

### 3.7. Molecular Docking of PA with Keap1 Protein

Molecular docking techniques are mainly employed to evaluate the stability and affinity of small target molecules bound to protein structures [27]. In molecular docking, it is generally believed that the lower the numerical value of the binding energy (kcal/mol) of the targeted small molecule to the interest protein, the stronger the binding ability and the more stable the docking system formed.

**Figure 6:**
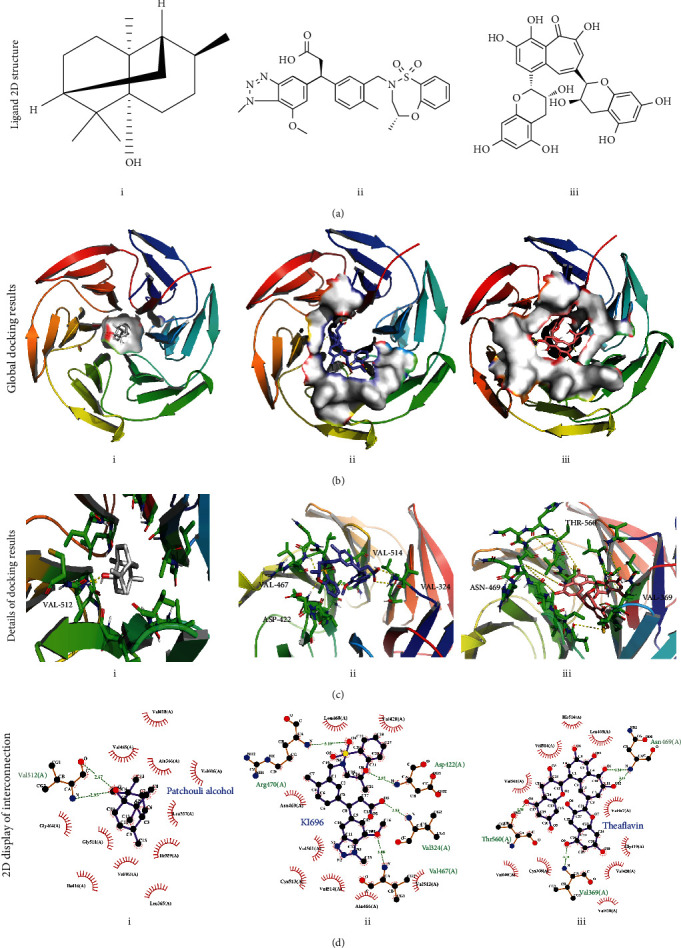
Docking between Nrf2 (1X2J) and three ligands (patchouli alcohol (i), KI696 (ii), and theaflavin (iii)). (a) Molecular structure diagram of patchouli alcohol, KI696, and theaflavin. (b) Global graph of docking results. (c) Local details of 3D molecular docking results. (d) The 2D display of molecular docking details.

In this study, molecular docking analysis was conducted in different small molecule drugs (PA, KI-676, and theaflavin) and Keap1 protein (1X2J) to explore the competence of PA against oxidative stress [25]. Among these ligands, KI-696 is a chemically synthetic Keap1 inhibitor, and theaflavin is reported as a natural Keap1/Nrf2/HO-1 regulator in chondrocytes [26, 28]. According to the results from Table 3, KI-696 had the lowest binding energy (-9.09) with Keap1, and PA (-7.73) had better binding energy than theaflavin (-7.48). Moreover, PA had better inhibit efficiency with Keap1 than KI-696 and theaflavin, while inhibit constant of KI-696 was better than PA with the order of KI696>PA>theaflavin (Table 3). Further, the 3D and 2D images clearly showed interactions between above ligands and Keap1 (Figures 6(b)–6(d)). Although there were more hydrogen bonds in KI-696 and theaflavin, there were more hydrophobic bond between PA and the residues around, including Leu365, Ala366, Ile416, Val418, Val463, Gly464, Val465, Gly511, Leu557, Ile559, and Val606 (Figure 6(d)). In brief, PA, KI-676, and theaflavin all have nice affinity with Keap1 protein. Although chemically synthetic KI-696 possesses the best binding ability, PA is more effective nature-derived inhibitor of Keap1 than theaflavin, suggesting that PA might exert better chondrocyte protective effect than theaflavin.

## 4. Discussion

A D-gal-induced subacute senescence model characterized by excessive ROS production, immune responses, behavioral disorders, and reduced antioxidant enzyme activity has been widely accepted to evaluate the effects of natural products on senescence [29]. When concentrations of D-gal in cells surpassed the extreme limit of catalytic and metabolic, D-gal could lead to cell permeability and swelling, accompanied by a large number of free radicals generation, thereby resulting in systemic aging [30]. PA is a naturally occurring tricyclic sesquiterpene found in the *Pogostemon cablin*. Several studies indicated that PA possesses pharmacological properties such as anticancer, neuroprotective, anti-influenza, anti-inflammatory, and antioxidant stress activities [31–35]. Nevertheless, antisenescence activity and the potential antisenescence mechanism of PA have not been elucidated. In this study, we report that PA showed an antisenescence activity associated with an upregulation of the Nrf2/HO-1 pathway, subsequently inhibiting oxidative stress induced by D-gal in chondrocytes and alleviating age-related cartilage quality decline.

The organ index is an important indicator applied to estimate viscera function from a macroperspective. It is commonly thought that senescence could induce organ atrophy, such as the liver, kidneys, spleen, and thymus [36]. As expected, the mice injected with D-gal had obviously lower organ indices of the liver, kidneys, spleen, and thymus than those of the NOR group, and PA (20 mg/kg) treatment alleviated this global organ dysfunction to some extent.

A decline in physical function is a major manifestation of aging, including reduced athletic capacity due to poor cartilage quality. In fact, the natural decline in cartilage performance with advancing age may be a perplexing phenomenon, and chronic age-related cartilage defects in middle-aged patients are perhaps an expression of early osteoarthritis (OA) [37, 38]. It is reported that the largest natural decrease of cartilage quality in normative IKDC data occurred between the ages of 51 and 65 years, followed by the decline between the ages of 35 and 50 years [39]. With the increase of age, chondrocytes, like other organs, the only cell type of cartilage, inevitably undergo senescence. Cellular senescence is often triggered by complex factors, such as telomere shortening, genome damage, oxidative stress response, and mechanical damage, among which oxidative stress overreaction is closely connected with age-related changes [40]. With reference to the previous studies, the excessive ROS accumulation would lead to the gradual decline of the proliferation and differentiation ability and physiological functions of chondrocytes, activate catabolic enzymes such as matrix metalloproteinases (MMPs) to remodel ECM, and stimulate downstream signaling pathways related to aging, including the p19^Arf-Mdm2^-p53-p21^Cip1/Waf1^ pathway and the p16^INK4a^-retinoblastoma (Rb) pathway [41–44]. As chondrocytes age, they synthesize smaller, less uniform proteoglycan molecules and less functional link proteins, and their mitotic and synthetic activities decline, which means that ECM homeostasis is broken down and cartilage quality would be dramatically reduced [45]. In the early stages of age-related degeneration of articular cartilage, the histopathological changes in articular cartilage are often characterized by decreased chondrocytes, a reduction in stress stimulation and anabolism, and a lessened secretion of active components of the cartilage matrix [46, 47]. In the present study, we observed histological changes of early cartilage degeneration in D-gal-induced aging mouse models and identified marker genes related to aging (Tp53, Cdkn1a/p21^Cip1/Waf1^, and Cdkn2a/p16^INK4a^) and ECM degradation (Col2a1, Mmp13, and Acan) by PCR or immunohistochemistry. In a senescence model induced by D-gal *in vitro*, we further ascertained the role of D-gal in inducing ECM homeostasis imbalance and chondrocyte senescence. Moreover, PA treatment relieved D-gal-induced aging cartilage damage both *in vitro* and *in vivo* experiments.

Nrf2, as a transcription factor whose expression decreases with age, is often involved in regulating the production of antioxidant substances (such as SOD, CAT, and GSH) [48, 49]. Under normal physiological conditions, the combination of Keap1 to Nrf2 protein inhibits Nrf2/HO-1 pathway, while Nrf2 dissociates and translocates into the nucleus to promote the expression of downstream genes after the cells were exposed to oxidative stress. Many previous studies have demonstrated that Nrf2 could directly or indirectly exert antioxidant stress and antiaging function [50–52]. Hence, it is reasonable to speculate that PA might play a role in antioxidant stress and antisenescent cartilage damage in D-gal-induced aging model by activating the Nrf2/HO-1 pathway. Xiao et al. found that treatment of PA diminishes heat-shock-induced oxidative stress in IEC-6 cells by regulating Nrf2/HO-1 pathway [21]. Our results proved that PA inhibited the oxidative stress response and alleviated the degradation of ECM components by revitalizing Nrf2 in D-gal aging mice. Besides, under the same condition of PA (10 *μ*M) intervention, the cell viability, antioxidant stress and antiaging ability, and ECM homeostasis of the Con-siRNA group were significantly restored, while Nrf2 knockdown cells were not strikingly recovered. These findings further suggested that the protective effect on aging cartilage damage is partially derived from activating Nrf2 to suppress the oxidative stress response in D-gal-induced mouse chondrocytes.

Molecular docking is a computer simulation algorithm for small molecules and proteins to recognize each other through geometric accouplement and energy matching [53]. Among the multitudinous docking analysis indicators, the binding energy is an important index for evaluating the stability of the results of docking. In previous researches, theaflavin and KI-696 could bind to the active center of Keap1 protein to promote the separation of Nrf2 protein from Keap1 protein, which enters the nucleus and activates the expression of antioxidant genes [26, 28]. In this study, molecular docking results showed that both theaflavin and PA were bound to the active center of Keap1 through intermolecular interaction and the binding energy of PA was lower, suggesting that PA may have the better activity than theaflavin. Hence, it is reasonable to think that activation of Nrf2 signaling pathway is one of the pathways which PA exerts an antisenescence role.

In conclusion, this study reveals that PA had protective effect on senescent chondrocytes and aging model induced by D-gal. It is suggested that the undying mechanism is through activation of the Nrf2/HO-1 pathway to alleviate oxidative stress in senile cartilage. Our study provides a promising treatment for preventing quality loss of senile cartilage through inhibiting oxidative stress-mediated senescence in chondrocytes.

## Figures and Tables

**Table 1:** List of primer sequences for RT-qPCR.

Gene	Forward primer	Reverse primer
Col2a1	5′-GGAATTTGGTGTGGACATAGGG-3′	5′-GGTCAGGTCAGCCATTCAGT-3′
Mmp13	5′-AGTTGACAGGCTCCGAGAAA-3′	5′-GGCACTCCACATCTTGGTTT-3′
Nrf2	5′-GTAGATGACCATGAGTCGCTTGCC-3′	5′-CTTGCTCCATGTCCTGCTCTATGC-3′
Hmox1	5′-ACCGCCTTCCTGCTCAACATTG-3′	5′-CTCTGACGAAGTGACGCCATCTG-3′
Sod1	5′-CCACTGCAGGACCTCATTTT-3′	5′-CACCTTTGCCCAAGTCATCT-3′
Cat	5′-AATATCGTGGGTGACCTCAA-3′	5′-CAGATGAAGCAGTGGAAGGA-3′
Gss	5′-CAGCCAGAACCCAGCCTTCC-3′	5′-GCGATTCAGGCCCAGGAACA-3′
Keap1	5′-CAACTTCGCTGAGCAGATTGGC-3′	5′-TGATGAGGGTCACCAGTTGGCA-3′
Nqo1	5′-CTCGTAGCAGGATTTGCC-3′	5′-GAAGCCACAGAAACGCA-3′
Tp53	5′-GCATGAACCGCCGACCTATCC-3′	5′-CCCAGGGCAGGCACAAACAC-3′
Cdkn1a/p21^Cip1/Waf1^	5′-TCCTGGTGATGTCCGACCTGTTC-3′	5′-CGGCGCAACTGCTCACTGTC-3′
Cdkn2a/p16^INK4a^	5′-GTCGCAGGTTCTTGGTCAC-3′	5′-TCTGCACCGTAGTTGAGCAG -3′
Acan	5′-TAGAGGATGTGAGTGGTCTT-3′	5′-TCCACTAAGGTACTGTCCAC-3′
Gapdh	5′-GAGTCAACGGATTTGGTCGT−3′	5′-TTGATTTTGGAGGGATCTCG−3′

Col2a1, collagen 2a1; Mmp13, matrix metalloproteinase 13; Nrf2, nuclear factor-erythroid 2-related factor 2; Hmox1, heme oxygenase-1; Sod1, superoxide dismutase 1; Cat, catalase; Gss, glutathione synthetase; Keap1, Kelch-like ECH-associated protein 1; Nqo1, NAD(P)H quinone dehydrogenase 1; Tp53, tumor protein p53; Cdkn1a/p21^Cip1/Waf1^, Cyclin-Dependent Kinase Inhibitor 1A; Cdkn2a/p16^INK4a^, Cyclin-Dependent Kinase Inhibitor 2A; Acan, aggrecan; Gapdh, glyceraldehyde phosphate dehydrogenase gene.

**Table 2:** The effect of patchouli alcohol (PA) on mental state, body weight, and organ indices.

Group	Body weight (g)	Organ index (mg/g)			
		Thymus index	Liver index	Spleen index	Kidney index
NOR	37.71 ± 5.48	5.02 ± 1.55	70.85 ± 3.73	7.78 ± 1.2	22 ± 2.84
D-gal	27.08 ± 3.87^∗^	1.7 ± 0.69^∗^	64.74 ± 3.07^∗^	5.74 ± 0.82^∗^	17.65 ± 3.23^∗^
D-gal and PA	31.62 ± 2.98^#^	2.36 ± 0.54^#^	69.83 ± 4.11^#^	7.24 ± 1.25^#^	20.76 ± 3.42^#^

Data are presented as mean ± SD from each group (*n* = 5, mean ± SD). ^∗^*P* < 0.05 vs. NOR group; ^#^*P* < 0.05 vs. D-gal group.

**Table 3:** Docking information between small molecules and proteins.

Ligands	Binding energy (kcal/mol)	Ligand efficiency	Inhibit constant	Electrostatic energy	Hydrogen bond	Hydrophobic bond
Patchouli alcohol	-7.73	-0.48	2.17 *μ*M	-0.08	1	11
Theaflavin	-7.48	-0.18	3.29 *μ*M	-0.37	4	10
KI-696	-9.09	-0.23	216.18 nM	-1.42	4	8

## Data Availability

The [DATA TYPE] data used to support the findings of this study are available from the corresponding author upon request.
